# Neglecting Long-Term Risks: Self-Disclosure on Social Media and Its Relation to Individual Decision-Making Tendencies and Problematic Social-Networks-Use

**DOI:** 10.3389/fpsyg.2020.543388

**Published:** 2020-10-27

**Authors:** Sina Ostendorf, Silke M. Müller, Matthias Brand

**Affiliations:** ^1^Department of General Psychology: Cognition and Center for Behavioral Addiction Research (CeBAR), University of Duisburg–Essen, Duisburg, Germany; ^2^Erwin L. Hahn Institute for Magnetic Resonance Imaging, Essen, Germany

**Keywords:** dual-process, decision making, social media, social-networks-use disorder, privacy, self-disclosure

## Abstract

Social media including social-networking sites (SNS) encourage people to disclose personal information via profiles and posts. It is assumed that positive short-term effects and immediate feedback (e.g., getting Likes) have a rewarding nature and may complicate the rational weighing of possible negative long-term consequences related to self-disclosure. Dual-process theories assume risky behaviors to result from more impulsive/short-term oriented compared to reflective/long-term oriented decision making. The current laboratory study investigates whether the extent of online self-disclosure is explained by the general tendency to choose short-term rewards by neglecting long-term risks as well as by tendencies toward a problematic social-networks-use. Participants (*N* = 88) were asked to log into their Facebook account to answer questions about their actual self-disclosing behavior. Furthermore, they performed an experimental decision-making task and answered a questionnaire assessing problematic social-networks-use. The quantity of self-disclosure via posts was negatively associated with advantageous decision making and positively with tendencies toward a problematic social-networks-use. The findings indicate that high self-disclosure via posts is associated with a general tendency to neglect long-term risks. Moreover, a problematic social-networks-use can additionally increase individual’s self-disclosure via posts.

## Introduction

The rapid development of technology has changed human lives in a variety of ways. Interpersonal communication nowadays takes exceedingly place in online environments due to an almost unrestricted, time- and location-independent accessibility and ubiquity enabled by mobile devices. Social networks, (micro-) blogs, and instant messaging services equip their users with several features to communicate and disclose content, for instance by sharing photos, videos or status updates with friends or the public. These platforms have therefore gained great importance for individuals to fulfill personal needs (e.g., Huang et al., 2014; Wegmann et al., 2017). However, personal information is also very attractive for companies, politicians, economists, and even criminals, as this sensitive data can be used for their own purposes. Furthermore, social media are often used as an instrument for various damaging behaviors such as engaging in cyberbullying, threatening or even stalking other people (e.g., Debatin et al., 2009; Aharony, 2016). Providing personal and sensitive information can therefore have several negative consequences for the individual user, which differ in the level of perceptibility and riskiness. Many individuals, however, still engage in social media excessively, providing even more information than necessarily demanded despite possible negative consequences (cf. Barth and de Jong, 2017). Even if users seem to be afraid that their online privacy might not reliably be safeguarded against privacy violations (Hoy and Milne, 2010; Yao, 2011) and even if privacy concerns were in some cases negatively related to online disclosures (e.g., Dwyer et al., 2007), the majority of studies agrees that privacy-related behaviors online cannot validly be predicted by privacy concerns (e.g., Acquisti and Gross, 2006; Tufekci, 2008; Zafeiropoulou, 2014). This gap between concerns and privacy-related behaviors is also referred to as the *privacy paradox* (see Barth and de Jong, 2017).

Yet, the reasons for this paradoxical behavior are not sufficiently understood. Previous work examined privacy-related decisions on social media mainly from a social-psychological, media-psychological, or information science perspective, but little is known about underlying cognitive processes. We therefore address the topic of privacy on social media from the perspective of cognitive psychology and investigate whether general decision-making tendencies are related to the disclosure of personal information. Moreover, individuals with a tendency to overuse social media applications might be vulnerable to disclose much information (Grammenos et al., 2017). Given the rewarding but also risky nature of sharing content on social media and the nearly infinite accessibility of such applications, it is especially important to investigate the relationships between actual disclosing behavior and general decision-making tendencies potentially underlying this behavior as well as tendencies toward a problematic use of social networks.

### Self-Disclosure

Following early studies, human conversations encompass particularly the sharing of private experiences, personal relationships, and individual opinions (e.g., Landis and Burtt, 1924; Emler, 1990). Furthermore, Tamir and Mitchell (2012) suggested “that humans so willingly self-disclose because doing so represents an event with intrinsic value, in the same way as with primary rewards such as food or sex” (p. 8041). Following this, such value may derive from (a) the possibility to introspect about the self and (b) to share this information with others. This assumption was supported by neural responses during self-disclosures: Both aspects solidly activated neural regions associated with reward processing, such as the nucleus accumbens and the ventral tegmental area, which are both part of the mesolimbic dopamine system (Tamir and Mitchell, 2012).

Regarding the online context, self-disclosure can be defined as a process of providing and communicating personal information to others through the Internet (Taddicken, 2014; Masur, 2018), while this can be done both rather reflectively but also impulsively. On social media, individuals can self-disclose particularly by sharing personal content via posts or by providing information on their individual profiles. While posts are frequent activities, profile updates are done less regularly (Strater and Lipford, 2008). However, in both cases, sharing personal content can be realized by just a few clicks. Further, a large proportion of posts on social media present own immediate experiences and other personal information (e.g., Naaman et al., 2010; Marshall et al., 2015). By sharing personal aspects, users can experience various benefits, such as maintaining relationships or building new ones, presenting oneself, or experiencing social support (e.g., Ellison et al., 2011; Taddicken and Jers, 2011; Cheung et al., 2015).

Since Facebook is one of the most popular SNS, it has been the target of several cyber-attacks varying in their degree of damage and leading to the necessity of improving possibilities that safeguard users’ personal data. Users themselves can for example untag photos or utilize different setting options to regulate and determine other people’s access to own personal data. However, individuals still seem to protect their personal information deficiently by ignoring potential long-term risks or underestimating their likelihood of occurrence (e.g., Debatin et al., 2009; Aivazpour et al., 2017; Barth and de Jong, 2017; Díaz Ferreyra et al., 2019). Possible long-term risks include for example identity theft, sexual harassment, cyberstalking, and commercial or criminal exploitation (e.g., Debatin et al., 2009; Walrave et al., 2012; Aharony, 2016). Furthermore, only limiting the visibility of personal information to specific people might protect one’s horizontal privacy to a certain extent (e.g., toward friends, co-workers), but one’s vertical privacy (e.g., protecting the information from being used by the platform or third parties; Bartsch and Dienlin, 2016; Quinn and Epstein, 2018) might still be violated. Thus, by providing a lot of personal information on SNS, individuals cannot only experience negative short-term consequences including sexting, negative feedback from others, or cybermobbing (Aharony, 2016) but also negative long-term consequences such as identity theft or criminal exploitation, which can even be more severe (Debatin et al., 2009; Aharony, 2016). In addition, especially negative consequences that can derive from the platform itself or third parties (vertical dimension), such as unintended commercial use of own data, are hardly avoidable, thus strengthening the need to better understand why individuals still share even more information than necessary.

### Privacy Conceptualizations and Theoretical Approaches to Related Online Behaviors

Based on Burgoon (1982), four privacy dimensions exist: informational, social, psychological, and physical privacy, whereby only the first three are defined to be relevant for the online context (Trepte and Reinecke, 2011; Dienlin and Trepte, 2015). Informational privacy describes one’s control over the extent, processing, and transferring of personal information. Social privacy encompasses access regulation in terms of proximity and distance to others, and psychological privacy describes the regulation of emotional and cognitive inputs and outputs and the intimacy of information (cf. Burgoon, 1982; Dienlin and Trepte, 2015). In the domain of Information Systems, research especially focuses on theories assuming that privacy decision making is a deliberate process (cf. Nyshadham and Van Loon, 2014), for example the Theory of Planned Behavior, the Theory of Reasoned Action, and the Protection Motivation Theory. The Protection Motivation Theory (Rogers, 1975) originally focused on health-related risks and at its core it is argued that based on a threat appraisal (evaluating and weighing a threat and anticipated rewards) and a coping appraisal (evaluating the possible protective response), individual’s protection motivation is shaped. Here, cognitive evaluation processes play a crucial role. Further, the Theory of Reasoned Action and the Theory of Planned Behavior (Ajzen and Fishbein, 1980; Ajzen, 1985) mainly argue that individual’s behavior is resulting from individual’s behavioral intention, which is shaped by the interplay of attitudes and subjective norms. In addition to that, the Theory of Planned Behavior includes individual’s perceived behavioral control. Here again, rather rational and controlled processes are expected to underlie human behavior. Such theories have been applied in the field of privacy on social media as well (e.g., Yao, 2011; Dienlin and Trepte, 2015). Further, many researchers use the notion of a *privacy calculus* (e.g., Dinev and Hart, 2006; Krasnova et al., 2010; Xu et al., 2011; Dienlin and Metzger, 2016) assuming that individuals make privacy decisions by trading off costs and benefits. Following this (and very close to the threat appraisal concept), self-disclosure is considered a rational choice, resulting in disclosing behavior if the expected gains exceed the anticipated potential risks (Debatin et al., 2009; Lee et al., 2013; Li et al., 2016).

However, research has pointed out that human decision making is not always rational, but also influenced by cognitive biases and heuristics (Kahneman and Tversky, 1979; Acquisti and Grossklags, 2007; Volz and Gigerenzer, 2012). Accordingly, the privacy calculus perspective or theories such as the Theory of Planned Behavior, which are based on the assumption of rationality (Turel and Qahri-Saremi, 2016) and miss the impulsive nature of decisions, might be too narrow to explain privacy-related behaviors. Thus, a theoretical lens is required that takes into account both rational as well as more impulsive processes.

### A Dual-Process Perspective for Privacy-Related Decisions on Social Media

Dual-process theories (e.g., Epstein, 2003; Evans, 2003; Bechara, 2005; Schiebener and Brand, 2015) assume that individuals’ decisions result from the interaction of strategic/reflective and intuitive/impulsive processes. These processes are assumed to stem from two neural systems, which are less strictly separated from each other, but rather interacting and thereby forming the final decision. The reflective system (also referred to as system 2 or rational-analytical system; Epstein et al., 1996; Kahneman, 2003) functions slow, serial, rule-guided, and cognitively controlled. Here, core assumptions of the formerly mentioned theories can be located (e.g., the rational weighing of perceived threats/risks and anticipated rewards/benefits). The impulsive system (also referred to as system 1 or the intuitive-experiential system; Epstein et al., 1996; Kahneman, 2003) enables fast and parallel processing based upon emotions and past experiences. The impulsive system is expected to process immediate gratification or punishment, while the reflective system is assumed to enable cognitive control over impulsive responses to achieve higher long-term goals (Bechara, 2005). Besides personal characteristics (e.g., impulsivity), the situation itself determines to what extent both systems are involved in the decision-making process (e.g., Schiebener and Brand, 2015). Based on the relative degree of uncertainty that a decision situation encompasses, either the impulsive or the reflective system can play a greater role: If at least part of the possible effects is known or can be estimated in a pending decision (meaning a moderate uncertainty is present), reflective processes are highly relevant for making advantageous choices. If the degree of uncertainty, however, increases, the impulsive system plays a greater role as relevant indications for strategic decisions are lacking (Starcke and Brand, 2012).

Dual-process assumptions have widely been applied to explain human behavior in different decision situations, including decisions under risk conditions (Schiebener and Brand, 2015), decisions under stress (Starcke and Brand, 2012) or intertemporal decisions (e.g., Metcalfe and Mischel, 1999). The latter assume that a “hot” system processes immediate outcomes, while “cool” cognitive processing is necessary to represent delayed/long-term consequences (Metcalfe and Mischel, 1999). A huge amount of literature exists on delay discounting describing that outcomes are devaluated if they are delayed in time (e.g., Madden and Johnson, 2010). Delay discounting tasks ask participants to choose a smaller reward now or a larger reward later. In this sense, the tendency to prefer smaller sooner rewards (i.e., steeper delay discounting) represents a facet of trait impulsivity that is linked to addictive behaviors (MacKillop et al., 2011; Amlung et al., 2017). However, choosing between a “smaller sooner” or a “larger later” option does not represent the fact that one choice option can have conflicting (short- and long-term) outcomes in itself. The focus of this paper is to apply the decision-making perspective to the area of online privacy-related decisions by using an experimental decision-making task that simulates decisions under risk conditions in which each option has conflicting short- and long-term consequences. In the context of online privacy, some authors already stressed the importance of automatic processes, bounded rationality, and emotions (John et al., 2011; Li et al., 2011; Kehr et al., 2013). Nyshadham and Van Loon (2014) argued that in privacy-related decision situations, there is an automatic and default assessment of risks, whereby “default risk judgments are based on automatic affect, *unless* they are endorsed/corrected/overridden by deliberation” (p. 4). Kehr et al. (2013) moreover argued that decisions in the context of online privacy illustrate a partially irrational process, which accentuates the role of affective and intuitive thinking. These considerations extend the perspective of a rather rational decision maker which is for instance at the core of the Theory of Planned Behavior (since it primarily focuses on goal-directed behaviors shaped by control beliefs and attitudes) or the privacy calculus assumption (assuming a rational weighing of risks and benefits). In sum, taking the theoretical lens of dual-process models, privacy-related decisions on social media can as well be guided by both reflective and impulsive processes.

When disclosing personal information, individuals can face possible short- and long-term outcomes that can be both positive and negative. For example, when posting a newly acquired garment via photo and comment, individuals can receive short-term acclaim (positive), but also dislike (negative) from others. Possible long-term outcomes can for example be strengthened relationships (positive), but also an increased vulnerability for unintended usage of one’s own content (negative). Besides the possibility that some users might primarily self-disclose due to expected positive long-term effects (e.g., popularity), we argue that most users of social networks self-disclose especially in prospect of short-term rewards including immediate gratification and support from their friends. Moreover, in both cases, evaluating possible risks, and especially long-term risks, can be challenging. We argue that short-term rewarding consequences (e.g., getting Likes for a photo) stay especially in conflict with potential long-term risks (e.g., commercial use of corresponding information) when individuals disclose personal information. As self-disclosing was found to offer high immediate gratification (Tamir and Mitchell, 2012), in particular the impulsive system seems to be triggered. However, choosing to disclose personal information more and more often can increase the likelihood of negative consequences in the long-run (mainly processed by the reflective system). As social media platforms are designed to fulfill personal needs and to support the experience of immediate gratification (Taddicken and Jers, 2011) while information about possible (negative) long-term consequences is lacking (Taddicken and Jers, 2011; Efroni et al., 2019), one may argue that the degree of uncertainty is increased and that the rewarding short-term consequences are more salient, resulting in decisions led more by impulsive rather than reflective processes. Furthermore, social media applications offer no immediate feedback about possible long-term consequences, which could moreover end up in an even greater disregard of long-term outcomes (Schiebener and Brand, 2015; Müller et al., 2017).

In addition, the design and cues displayed on social media bear the potential to influence privacy-related decisions. Nyshadham and Van Loon (2014) discuss that the website design can influence whether privacy decisions are more likely relying on cognitive ease (based on effortless impulsive processes) than on cognitive strain (based on effortful reflective processes). Trepte (2015) further stated that familiar cues on social media (*warm affordances*) encourage users to upload and provide personal content, whereas *cold affordances* (e.g., privacy conditions) are less familiar to users and differ in their immediacy and accessibility from warm affordances.

Taken together, both might hinder rational decision making on social media: On the one hand, specific cues, website affordances, and the prospect of immediate gratification could promote a predominance of the impulsive system resulting in shortsighted decisions, and on the other hand, lacking feedback about long-term (risky) consequences might increase uncertainty hindering the reflective system to override impulsive judgments. This decision-making tendency in an intense manner, namely a hyperactivity of the impulsive system, has further been associated with excessive/addictive behaviors (e.g., Bechara, 2005; Weinstein, 2017). Accordingly, a potential link between self-disclosure and problematic social-networks-use might exist.

### Online Self-Disclosure and Problematic Social-Networks-Use

Individuals are nowadays “permanently online and permanently connected” (Vorderer et al., 2016, p. 695). Further, the permanent access to social media applications can lead to an excessive engagement (Klimmt and Brand, 2017) and an increased usage can lead to a greater tendency toward self-disclosure (Walrave et al., 2012; Chang and Heo, 2014). Accordingly, it seems important to investigate whether the tendency toward an overuse/problematic use of social media applications might have an effect on the amount of self-disclosures, which in turn may increase the risk of negative long-term consequences. Following Andreassen and Pallesen (2014), a problematic/addictive social-networks-use can be defined as “being overly concerned about SNSs, to be driven by a strong motivation to log on to or use SNSs, and to devote so much time and effort to SNSs that it impairs other social activities, studies/job, interpersonal relationships, and/or psychological health and well-being” (p. 4054). During the last two decades, research on problematic use of social media applications has grown steadily, including authors that account this overuse as a clinical phenomenon (e.g., Montag et al., 2018; Wegmann et al., 2018a). Although the phenomenon has various terms, for example social-networks-use disorder (Montag et al., 2019; Wegmann and Brand, 2019; Wegmann et al., 2020), Internet-communication disorder (Montag et al., 2018; Wegmann et al., 2018a, b) or social networking addiction (Guedes et al., 2016), there is growing evidence for parallels to clinically relevant disorders including gaming disorder and gambling disorder (for a current review see Wegmann et al., 2018a).

A current model that has been applied frequently in the field of Internet-use disorders (IUD), the I-PACE model (Interaction of Person-Affect-Cognition-Execution; Brand et al., 2016, 2019), illustrates a theoretical framework for the development and maintenance of specific IUD. In this process model, interactions of variant personal predispositions and affective, cognitive as well as executive components are considered as relevant mechanisms for the development and maintenance of different types of IUD. To emphasize how the development of a problematic social-networks-use could increase individual’s self-disclosures, we draw on the argumentation of Brand et al. (2019) that in early stages of addictive tendencies, situational triggers on an internal (e.g., experiencing specific moods) or external level (e.g., being confronted with social media cues such as pop-up messages) may lead to cognitive and affective responses, for example in the form of increased attention toward these cues. This subsequently leads to the decision to behave in a specific way. This behavior can for instance encompass the posting of one’s current feelings or personal experiences such as being overly excited due to an upcoming trip. As the decision to use social media applications (e.g., Facebook) to post details of one’s personal life (thus engaging in self-disclosure) enables the experience of gratification (e.g., by receiving positive comments), subjective reward expectancies can subsequently increase and one’s individual coping style might be reinforced. In the following, finding oneself in similar situations with comparable external or internal triggers might increase the likelihood that one responds with enhanced desire or an increased anticipation of gratification, leading to the recurring decision to use specific applications and to self-disclose by presenting personal information online. This “inner circle” (Brand et al., 2019, p. 2) may become stronger as time passes by and consequently, individuals with an increasing tendency for an addictive social-networks-use might face the difficulty to inhibit their affective responses, leading to more impulsive/less controlled behavior including increased self-disclosures. Furthermore, specific triggers might become even more salient and specific behaviors might become more habitual and automatic over time. Besides, addiction-related stimuli were found to activate the ventral/dorsal striatum and further limbic structures associated with the brain’s reward system (Fauth-Bühler and Mann, 2017; Luijten et al., 2017), which is also triggered when disclosing personal information (Tamir and Mitchell, 2012).

### Research Questions

Based on the above mentioned theoretical considerations, we expect individuals with a general tendency to prefer short-term gratifications over long-term risks to be prone to disclose personal information online. Furthermore, we assume tendencies toward problematic social-networks-use to account for additional variance in online self-disclosure.

Hypothesis: Self-disclosure on social media is predicted by (a) the tendency to choose immediate gratification despite long-term risks and (b) the tendency toward problematic social-networks-use.

Referring back to the fact that the interplay of reflective and impulsive processes is assumed to be crucial for both a general preference for short-term rewarding options over long-term consequences and a tendency toward a problematic use of specific online applications (Schiebener and Brand, 2017), interactions between the two factors might be assumed. More precisely, it may be assumed that not only the general decision-making tendency solely effects online self-disclosure but especially if it is accompanied by a high tendency toward problematic social-networks-use. Accordingly, we further address the following research question:

RQ: Does the interplay between the general decision-making tendency and the tendency toward problematic social-networks-use further explain self-disclosure on social media?

As mentioned earlier, personal disclosures can be realized via posts and via profile information, which differ in their frequency of creation and adjustment (Strater and Lipford, 2008). Since posts provide many opportunities to socially interact with others and thus may enable the experience of gratification more than profile information, we test the hypothesis and subsequent research question separately for self-disclosure via posts and self-disclosure via profile information.

According to the previously mentioned characteristics of decisions to disclose personal information on social media, we especially focus on the decision-making behavior in situations with conflicting short- and long-term consequences in which no immediate feedback about long-term risks is perceivable. In order to measure self-disclosure more objectively than it is possible with common self-reports, we asked participants to give us concrete information on their actual behavior by logging into their private Facebook account. Facebook itself was chosen since it is the most popular social network worldwide (Statista, 2019b).

## Materials and Methods

### Participants and Recruitment

Eighty-eight participants (16–56 years, *M* = 25.61, *SD* = 6.70) took part in this study. Fifty-six were females, 31 males and one participant stated to be divers. Participating required to use Facebook actively, which was defined by having at least one posting (within one’s own Facebook Timeline) in the last 3 months. Beyond Facebook, 98.9% of all participants used WhatsApp, followed by Instagram (86.4%), and the Facebook Messenger (80.7%). Further applications including Snapchat, Twitter, iMessage, Skype or Threema were used by less than 40% of all participants. On average (self-reported estimates), WhatsApp was used 98.72 min (*SD* = 119.37) per day, followed by Instagram with 51.15 min (*SD* = 44.09), and Facebook with 34.24 min (*SD* = 26.81). Other applications were used less than 10 min per day. Regarding their occupation, 67 participants stated to be students, 11 were employees, three were job seekers, two were self-employed persons, two were pupils, one stated to be pensioner, and two did not reveal information on their occupation. The current sample was recruited at the University of Duisburg–Essen via notices on the campus, mailing lists, newspaper advertising, Facebook and other social networks. Students could choose between credit points or a remuneration of 10 Euro/hour for taking part. Non-students received 10 Euro/hour. In total, the laboratory study with an individual setting took about 90 min, starting with a written information about the procedure and a written informed consent given by the participants. In this course, the term “privacy” was not mentioned. Afterward, participants answered online questionnaires and performed a decision-making paradigm (the Cards and Lottery Task), followed by logging into Facebook with their private account (using a protected browser mode) to answer specific questions regarding their self-disclosures. The local Ethics Committee of the Department of Computer Science and Applied Cognitive Science of the University of Duisburg–Essen approved the study and it was conducted in accordance with the Declaration of Helsinki.

### The Cards and Lottery Task (CLT)

To assess the decision-making tendency in situations that contain conflicting short- and long-term consequences, the Cards and Lottery Task (CLT; Müller et al., 2017) was used. Participants played the CLT in the so-called *partial feedback* version, which simulates decision situations in which feedback is provided about the short-term consequences but not about the long-term prospects of a decision. In this computerized task, participants should try to win as much virtual money as possible. The task consists of two parts: first, participants choose 36 times between two decks of cards that vary from round to round. During this phase, participants collect virtual money on a short-term account. The second part comprises a lottery, in which an additional large amount can be won or lost leading to an overall long-term outcome. The risk of losing depends on the decisions made in the previous part of the game. For both card decks, explicit information is given on the cards contained in each deck. Each card has two properties: one representing short-term and one representing long-term consequences. Short-term consequences encompass immediate gains or losses (virtual money) and affect the short-term account. Long-term consequences are represented by symbols [bombs (negative), stars (positive), or no symbol (neutral)]. Bombs represent the risk of losing the lottery at the end (the more bombs, the higher the risk to lose), while stars represent the chance to win the lottery (the more stars the lower the risk to lose). The two decision options (i.e., decks of cards) both offer a conflict between short-term and long-term consequences: one deck contains cards with high immediate gains but, at the same time, many bombs (increasing the risk for negative long-term outcome), while the other deck contains cards with only low immediate gains or immediate losses but, at the same time, many stars (increasing the chance for a positive long-term outcome). Thus, the left deck is designed in the way that it is advantageous regarding the short-term account, but (on average) disadvantageous in the long run, whereas the right deck is advantageous in the long run, but (on average) disadvantageous regarding the short-term account balance.

**FIGURE 1:**
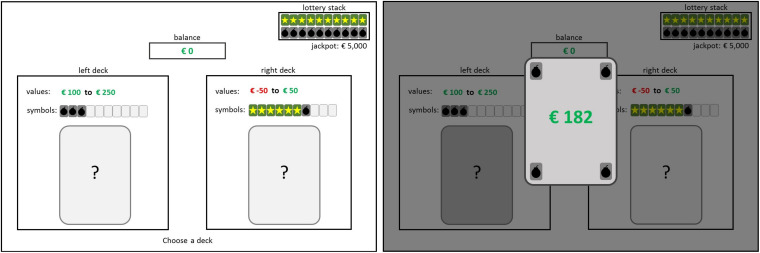
Example of a decision situation in the CLT with the respective feedback presentation in the full feedback version (training trials).

**FIGURE 2:**
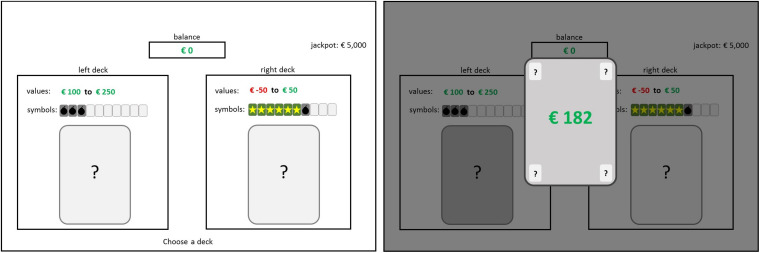
Example of a decision situation in the CLT with the respective feedback presentation in the partial feedback version.

After detailed instructions, participants perform five training trials in which feedback on both the short- (immediate gain or loss of virtual money) and the long-term consequence (star, bomb, or no symbol) is provided after each decision. Also, the actual proportion of collected stars/bombs is visualized in the corner of the screen (see Figure 1). After the training trials, the participants are informed that, from now on, the information about drawn symbols (i.e., long-term consequences) and the proportion of collected stars/bombs will be hidden (see Figure 2). Thus, participants only receive feedback on the immediate gain or loss associated with a card, but not whether the card dawn was a star-card, a bomb-card, or had no symbol (meaning no long-term effect) and how this affects the probability of winning or losing the lottery at the end.

To assess the task performance, Müller et al. (2017) propose different scores. Since the CLT *net score* best reflects the decision-making tendency we address in this study, we only use this score for testing the hypothesis. By calculating the number of choices of the long-term deck minus the number of choices of the short-term deck, participants receive an individual score with a lower value indicating a preference for short-term- over long-term-oriented decisions. In other words, a lower score reflects individual’s tendency to choose immediate gratification despite negative long-term consequences, thus representing a tendency for impulsive processing. Values of the *net score* range between −36 and 36. For a more detailed description of the task design, contingencies, and measures please see Müller et al. (2017).

### Short Internet Addiction Test Modified for Social-Networks-Use

To measure tendencies toward problematic social-networks-use, we used the short Internet Addiction Test (s-IAT) by Pawlikowski et al. (2013) in a modified version specified for social-networks-use (Wegmann et al., 2015; s-IAT-SNS). Participants rate their subjectively perceived complaints in everyday life due to the excessiveness of their social-networks-use. The term “use” is thereby explained to encompass both, the active (e.g., creating new content) as well as passive use (e.g., browsing and reading posts) of social networks. The scale includes twelve items rated from 1 = “never” to 5 = “very often.” The calculated sum score of the *s-IAT-SNS* ranges from 12 to 60, whereby scores >30 can be interpreted as being at risk, and scores >37 as showing a problematic usage (in accordance with Pawlikowski et al., 2013). The overall internal consistency was α = 0.855.

### Self-Disclosure on Social Media

To measure online self-disclosure, we asked participants to log into their private account on Facebook and to report their actual disclosures, which were in a first step assigned to three privacy dimensions derived from literature: an informational, psychological, and social dimension (cf. Burgoon, 1982; Krämer and Haferkamp, 2011; Dienlin and Trepte, 2015). Moreover, we applied these dimensions for disclosures via profile information and via posts. In more detail, the informational dimension in terms of profile information includes the veritable disclosure of aspects such as one’s birthday, current residence or phone number (see Table 1). The psychological dimension includes the veritable disclosure of for instance one’s personal relationship status, sexual orientation, or personal interests (see Table 1). For the social dimension, we asked the participants who had access to this information. Here, for each aspect that was disclosed they had to state whether this was visible “only for me,” “only for a few of my friends/certain friend lists,” “for all of my friends,” or “for everybody (the public).” These categories were derived from the setting options on Facebook.

**TABLE 1:** Overview of the study’s constructs and their respective operationalizations.

		Operationalized variables			
Construct	Instrument	Variable name (description)	Answer format	Score	Possible value range
Decision-making tendency	Cards and Lottery Task (CLT)	Net score (Choices of the left/short-term over the right/long-term deck)	−1 = “left deck,” 1 = “right deck” per trial	Sum score	−36 to 36
Problematic social-networks-use	Short Internet Addiction Test modified for problematic social-networks-use	S-IAT-SNS (Higher scores indicate higher tendencies toward problematic social-networks-use)	5 point Likert Scale (1 = “never” to 5 = “very often”)	Sum score	12–60
Self-disclosure – Profile	Facebook Log-in and questionnaire (profile)	Quantity of self-disclosure via profile (Question: “*Please check all the information you have truthfully provided on Facebook.*”)	0 = “not checked,” 1 = “checked” for 11 items^a^	Sum score	0–11
		Horizontal width of self-disclosure via profile^b^ (Question: “*Please indicate the visibility setting of each profile aspect.*”)			
		Profile aspects visible “only for me”	0 = “not checked,” 1 = “checked” for 11 items^a^	Sum score	0–11
		Profile aspects visible “for (certain) friends”	0 = “not checked,” 1 = “checked” for 11 items^a^	Sum score	0–11
		Profile aspects visible “for everybody (the public)”	0 = “not checked,” 1 = “checked” for 11 items^a^	Sum score	0–11
Self-disclosure – Posts	Facebook Log-in and questionnaire (posts)	Quantity of active involvement (Question: “*How often have you posted anything in the last 3 months?*”)	Individual number entry	Entered number	1 – unlimited^c^
		Quantity of self-disclosure via posts (Question: “*In how many of your last maximal ten posts did you provide information about*…”)	Individual number entry (0–10) for 6 items^d^	Sum score	0–60
		Horizontal width of self-disclosure via posts^e^ (Question: “*How many of your last maximal ten posts have the following visibility settings?*”)			
		Posts visible “only for me”	Individual number entry (0–10)	Entered number	0–10
		Posts visible “for (certain) friends”	Individual number entry (0–10)	Entered number	0–10
		Posts visible “for everybody (the public)”	Individual number entry (0–10)	Entered number	0–10

*^*a*^ Items: *Phone number*, *email address*, *birthday*, *current residence*, *current workplace/school/university* (informational dimension); *Personal relationship status*, *sexual orientation*, *religion/religious views*, *political views*, *personal interests (sports, music, movies, etc.) via Likes*, *life events* (psychological dimension).^*b*^ Represents the social dimension with regard to self-disclosures via profile. Selection is exclusive for one of the three visibility categories per item. The sum of all categories cannot exceed 11.^*c*^ In this study the maximum of entered numbers was 27.^*d*^ Items: …*what your concrete activity/event (e.g., going to the cinema, changing jobs, etc.) was?*, …*who has taken part in your activity/event (linking of people)?*, …*where you were at the time of the activity/event (e.g., city, venue, etc.)?* (informational dimension); …*what your attitude/personal opinion about something (e.g., on a political or religious level, etc.) was?*, …*what emotions you felt (e.g., joy, anger, etc.)?*, …*what experiences you made (e.g., during a trip)?* (psychological dimension).^*e*^ Represents the social dimension with regard to self-disclosures via posts. Number entry is exclusive for one of the three visibility categories. The sum of all categories does not exceed 10.*

Regarding disclosures via posts, we asked the participants how many posts they altogether had created during the last 3 months (see Table 1). After that, they were informed to only account for the last maximal ten posts and to state in how many of these they disclosed (i) *what* the activity/event was, (ii) *who* has taken part in the activity/event, and (iii) *where* they were at the time of the activity/event, representing the informational dimension. Further, they were asked in how many of the last maximal ten posts they disclosed (i) their *attitude/personal opinion* about something, (ii) the *emotions* they felt, and (iii) the *experiences* they made, representing the psychological dimension. For the social dimension, participants had to state how many of the last maximal ten posts we accessible “only for me,” “only for a few of my friends/certain friend lists,” “for all of my friends,” or “for everybody (the public).”

Participants received a short manual to ensure that they all knew where to navigate to find all information asked for. Furthermore, they could also ask the examiner at any time if they had further questions. For testing the hypotheses, we calculated in a second step the following scores (see Table 1): For disclosures in the course of profile information, we use (a) the *quantity of self-disclosure via profile* by adding up the number of truthfully disclosed aspects on the informational and psychological dimension (values can range between zero and eleven) and (b) the *horizontal width of self-disclosure via profile* by adding up how many aspects were provided either “only for me,” “for (certain) friends,” or “for everybody (the public)” with each category containing values between zero and eleven, whereby the sum of all categories does not exceed eleven. For the *quantity of self-disclosure via profile*, we included both the informational and psychological dimension since they have in common to depict what is disclosed, in contrast to the social dimension that depicts to whom the information is disclosed. Regarding the *horizontal width of self-disclosure via profile*, we additionally merged the former categories “only for a few of my friends/certain friend lists” and “for all of my friends” to have three more distinct categories.

For disclosures via posts, we use (a) the *quantity of active involvement* which is the amount of posts during the last 3 months, (b) the *quantity of self-disclosure via posts* by adding up in how many of the last maximal ten posts participants disclosed aspects on the informational and psychological dimension (values can range between zero and 60 as each of the six aspects *what*, *who*, *where*, *attitude/personal opinion*, *emotions*, and *experiences* can be included in the maximum of ten posts), and (c) the *horizontal width of self-disclosure via posts* by adding up how many of the last maximal ten posts were visible either “only for me,” “for (certain) friends,” or “for everybody (the public)” (see Table 1). Here, we again combined the informational and psychological dimension and also merged the former categories “only for a few of my friends/certain friend lists” and “for all of my friends.” Each of the three remaining categories of the *horizontal width of self-disclosure via posts* can contain values between zero and ten, whereby the sum of all categories does not exceed ten.

### Overview of Constructs and Respective Measures

Table 1 summarizes all constructs of this study and their corresponding operationalizations.

### Statistical Analyses

For the statistical analyses we used SPSS 24.0 for Windows (IBM, 2017). To test for bivariate correlations we calculated Pearson’s correlations where a coefficient |r| ≥ 0.10 indicates a small, |r| ≥ 0.30 a medium, and |r| ≥ 0.50 a large effect (Cohen, 1988). We further calculated hierarchical moderated regression analyses to (a) test for the predictive power of decision-making tendencies and problematic social-networks-use on self-disclosure and (b) to address the subsequent research question of whether the interaction of both predictors might provide further explanation of the dependent variable’s variance (with Fisher’s z-transformed independent variables). With *N* = 88 participants, the calculated power analysis using G^∗^Power (version 3.1.9.2) revealed a power of 0.86 given a medium effect size for multiple regression analyses [*f*^2^ = 0.15; based on Cohen (1988)]. To detect a medium effect size with a power of 0.80 (Cohen, 1988), a sample of at least *N* = 77 would have been necessary.

## Results

### Descriptive Values and Multivariate Statistics

Mean values and standard deviations of all variables are depicted in Table 2. On average, individuals tended to prefer long-term choices (indicated by the positive CLT *net score*). According to the cut-offs proposed for the s-IAT (Pawlikowski et al., 2013) about 53% of all participants showed a functional, 34% were at risk, and 13% showed a problematic social-networks-use. Furthermore, participants created on average about five posts in the last 3 months, disclosed about four aspects in the last maximal ten posts and made their posts mostly visible “for (certain) friends” or “for everybody (the public)” (see Table 2). Furthermore, they disclosed on average five out of eleven aspects within their profile information and these aspects were mostly visible “for (certain) friends,” followed by “for everybody (the public)” (Table 2). Table 3 shows bivariate correlations between all variables. Regarding self-disclosures via profile, we found no significant correlations with decision making (CLT *net score*) or problematic social-networks-use (*s-IAT-SNS*). However, the CLT *net score* was significantly negatively associated with the *quantity of active involvement* and the *quantity of self-disclosure via posts* (with small to medium effect sizes). In addition, the *s-IAT-SNS* was significantly positively related to the *quantity of self-disclosure via posts* (with a small to medium effect). For the *horizontal width of self-disclosure via posts*, we found no significant correlations.

**TABLE 2:** Mean values, standard deviations, and range of the study’s variables.

	*M*	*SD*	Range
**Decision-making tendency**			
CLT net score	2.55	14.98	−36 to 34
**Problematic social-networks-use**			
S-IAT-SNS	29.42	7.33	13–44
**Self-disclosure – Profile**			
Quantity of self-disclosure via profile	5.10	1.93	1–9
Horizontal width of self-disclosure via profile			
Profile aspects visible “only for me”	0.95	0.91	0–4
Profile aspects visible “for (certain) friends”	2.52	1.69	0–8
Profile aspects visible “for everybody (the public)”	1.59	1.76	0–7
**Self-disclosure – Posts**			
Quantity of active involvement^*a*^	4.69	5.65	1–27
Quantity of self-disclosure via posts^*b*^	3.59	4.18	0–20
**Horizontal width of self-disclosure via posts^*b*^**			
Posts visible “only for me”	0.05	0.21	0–1
Posts visible “for (certain) friends”	1.94	2.63	0–10
Posts visible “for everybody (the public)”	1.76	2.43	0–10

*CLT net score = number of choices of the left/short-term over the right/long-term deck in the Cards and Lottery Task; S-IAT-SNS = sum score of the Short Internet Addiction Test modified for social networks.^*a*^During the last 3 months.^*b*^Corresponding to the last maximal ten posts.*

**TABLE 3:** Bivariate correlations between all variables.

Variable	2	3	4	5	6	7	8	9	10	11
(1) CLT net score	–0.078	0.016	–0.052	0.049	–0.022	−0.235*	−0.275**	–0.140	–0.161	–0.080
(2) S-IAT-SNS	–	–0.061	0.029	–0.070	–0.024	0.060	0.257*	–0.102	0.033	0.035
(3) Quantity of self-disclosure via profile		–	0.501**	0.431**	0.429**	0.169	–0.023	–0.097	0.065	0.032
(4) Profile aspects visible “only for me”^a^			–	0.083	–0.048	–0.063	–0.099	–0.170	–0.040	–0.036
(5) Profile aspects visible “for (certain) friends”^a^				–	−0.519**	–0.079	–0.007	–0.003	0.175	–0.126
(6) Profile aspects visible “for everybody (the public)”^a^					–	0.302**	0.043	–0.011	–0.080	0.189
(7) Quantity of active involvement^b^						–	0.706**	–0.105	0.536**	0.471**
(8) Quantity of self-disclosure via posts^c^							–	–0.084	0.440**	0.424**
(9) Posts visible “only for me”^c,d^								–	–0.100	–0.136
(10) Posts visible “for (certain) friends”^c,d^									–	−0.335**
(11) Posts visible “for everybody (the public)”^c,d^										–

CLT net score = number of choices of the left/short-term over the right/long-term deck in the Cards and Lottery Task; S-IAT-SNS = sum score of the Short Internet Addiction Test modified for social networks.*p ≤ 0.05 (two-tailed).**p ≤ 0.01 (two-tailed).^*a*^Corresponding to the *horizontal width of self-disclosure via profile*.^*b*^During the last 3 months.^*c*^Corresponding to the last maximal ten posts.^*d*^Corresponding to the *horizontal width of self-disclosure via posts*.

### Testing the Hypothesis and Research Question

In accordance with our hypothesis and research question, we calculated hierarchical moderated regression analyses with the CLT *net score* in a first step, the sum score of the *s-IAT-SNS* in a second step and the interaction term in the third step. As dependent variable we used (for disclosures via profile information) the *quantity of self-disclosure via profile* and the *horizontal width of self-disclosure via profile* (represented by the number of aspects that were provided for each category). For disclosures via posts, we used the *quantity of active involvement*, the *quantity of self-disclosure via posts*, and the *horizontal width of self-disclosure via posts* (represented by the number of posts that were visible for each category) as dependent variable. The results of the different models and corresponding statistical values including beta-coefficients can be found in Tables 4, 5.

**TABLE 4:** Results and corresponding regression coefficients of the hierarchical moderated regression analyses explaining self-disclosure via posts.

	Statistical values (stepwise)			Regression coefficients of the overall model					Statistical values of the overall model
	*R*^2^/Δ*R*^2^	*F*/Δ*F*	*p*	*B*	*SE (B)*	β	*t*	*p*	
**Model 1 (criterion *quantity of active involvement*^a^)**									
CLT net score	0.055	5.05	**0.027**	−0.09	0.04	−0.233	−2.19	**0.031**	
S-IAT-SNS	0.002	0.16	0.694	0.03	0.08	0.044	0.41	0.681	
Interaction	0.000	0.04	0.850	−0.00	0.00	−0.020	−0.19	0.850	*R*^2^ = 0.058, *F*(3,84) = 1.71, *p* = 0.171
**Model 2 (criterion *quantity of self-disclosure via posts*^b^)**									
CLT net score	0.076	7.05	**0.009**	−0.07	0.03	−0.261	−2.56	**0.012**	
S-IAT-SNS	0.056	5.49	**0.021**	0.14	0.06	0.247	2.42	**0.018**	
Interaction	0.007	0.66	0.419	−0.00	0.01	−0.083	−0.81	0.419	*R*^2^ = 0.139, *F*(3,84) = 4.50, ***p* = 0.006**
**Model 3 (criterion *posts visible “only for me*”^b,c^)**									
CLT net score	0.020	1.72	0.194	−0.00	0.00	−0.148	−1.37	0.174	
S-IAT-SNS	0.013	1.13	0.290	−0.00	0.00	−0.117	−1.08	0.284	
Interaction	0.001	0.05	0.823	0.00	0.00	0.024	0.22	0.823	*R*^2^ = 0.033, *F*(3,84) = 0.96, *p* = 0.417
**Model 4 (criterion *posts visible “for (certain) friends”*^b,c^)**									
CLT net score	0.026	2.29	0.134	−0.03	0.02	−0.157	−1.45	0.150	
S-IAT-SNS	0.000	0.04	0.849	0.01	0.04	0.014	0.13	0.900	
Interaction	0.003	0.28	0.597	0.00	0.00	0.057	0.53	0.597	*R*^2^ = 0.030, *F*(3,84) = 0.85, *p* = 0.468
**Model 5 (criterion *posts visible “for everybody (the public)”*^b,c^)**									
CLT net score	0.006	0.55	0.460	−0.01	0.02	−0.076	−0.70	0.486	
S-IAT-SNS	0.001	0.07	0.792	0.01	0.04	0.026	0.23	0.816	
Interaction	0.001	0.06	0.810	0.00	0.00	0.026	0.24	0.810	*R*^2^ = 0.008, *F*(3,84) = 0.22, *p* = 0.881

*Significant values depicted in bold; CLT net score = number of choices of the left/short-term over the right/long-term deck in the Cards and Lottery Task; S-IAT-SNS = sum score of the Short Internet Addiction Test modified for social networks.^*a*^During the last 3 months.^*b*^Corresponding to the last maximal ten posts.^*c*^Corresponding to the *horizontal width of self-disclosure via posts*.*

**TABLE 5:** Results and corresponding regression coefficients of the hierarchical moderated regression analyses explaining self-disclosure via profile.

	Statistical values (stepwise)			Regression coefficients of the overall model					Statistical values of the overall model
	*R*^2^/Δ*R*^2^	*F*/Δ*F*	*p*	*B*	*SE (B)*	β	*t*	*p*	
**Model 1 (criterion *quantity of self-disclosure via profile*)**									
CLT net score	0.000	0.02	0.886	0.00	0.01	0.001	0.01	0.996	
S-IAT-SNS	0.004	0.30	0.583	−0.01	0.03	−0.034	−0.32	0.753	
Interaction	0.048	4.25	**0.042**	−0.01	0.00	−0.221	−2.06	**0.042**	*R*^2^ = 0.052, *F*(3,84) = 1.53, *p* = 0.213
**Model 2 (criterion *profile aspects visible “only for me”*^a^)**									
CLT net score	0.003	0.24	0.629	−0.00	0.01	−0.051	−0.47	0.643	
S-IAT-SNS	0.001	0.05	0.819	0.00	0.01	0.026	0.24	0.812	
Interaction	0.000	0.01	0.920	0.00	0.00	−0.011	−0.10	0.920	*R*^2^ = 0.003, *F*(3,84) = 0.10, *p* = 0.961
**Model 3 (criterion *profile aspects visible “for (certain) friends”*^a^)**									
CLT net score	0.002	0.20	0.653	0.00	0.01	0.037	0.34	0.735	
S-IAT-SNS	0.004	0.38	0.541	−0.01	0.03	−0.050	−0.46	0.649	
Interaction	0.020	1.77	0.187	−0.00	0.00	−0.144	−1.33	0.187	*R*^2^ = 0.027, *F*(3,84) = 0.76, *p* = 0.506
**Model 4 (criterion *profile aspects visible “for everybody (the public)”*^a^)**									
CLT net score	0.000	0.04	0.839	−0.00	0.01	−0.029	−0.27	0.790	
S-IAT-SNS	0.001	0.06	0.813	−0.00	0.03	−0.013	−0.12	0.907	
Interaction	0.012	1.02	0.315	−0.00	0.00	−0.110	−1.01	0.315	*R*^2^ = 0.013, *F*(3,84) = 0.37, *p* = 0.773

*Significant values depicted in bold; CLT net score = number of choices of the left/short-term over the right/long-term deck in the Cards and Lottery Task; S-IAT-SNS = sum score of the Short Internet Addiction Test modified for social networks.^*a*^Corresponding to the *horizontal width of self-disclosure via profile.**

#### Results for Self-Disclosures via Posts as Dependent Variable

The results (see Table 4) revealed a significant effect of the *net score* on the *quantity of active involvement* (*R*^2^ = 0.055, *F* = 5.05, *p* = 0.027) and the *quantity of self-disclosure via posts* (*R*^2^ = 0.076, *F* = 7.05, *p* = 0.009). The corresponding beta-coefficients were negative, indicating negative relationships (Table 4). Further, the *net score* did not reveal a significant effect on the *horizontal width of self-disclosure via posts*: neither for the number of posts visible “only for me,” “for (certain) friends” nor “for everybody (the public).”

Referring to the *s-IAT-SNS* sum score, we found an additional significant main effect on the *quantity of self-disclosure via posts* (Δ*R*^2^ = 0.056, Δ*F* = 5.49, *p* = 0.021) with a corresponding positive beta-coefficient (Model 2, Table 4). The *s-IAT-SNS* sum score was not a significant predictor for the *quantity of active involvement* or the *horizontal width of self-disclosure via posts* (applying for any category).

Regarding the third step (representing the research question), the results revealed no significant interaction effects (Table 4). For self-disclosure via posts, all Δ*R*^2^ ≤ 0.007, all Δ*F* ≤ 0.66, and all *p* ≥ 0.419. Finally, the overall regression model with the *net score* and the *s-IAT-SNS* sum score as predictors (Model 2, Table 4) for the *quantity of self-disclosure via posts* was significant with both predictors explaining 13.2% of the criterion’s variance [*R*^2^ = 0.139, *F*(3,84) = 4.50, *p* = 0.006].

Taken together, our hypothesis is partially supported when taking the *quantity of active involvement* as dependent variable and fully supported when taking the *quantity of self-disclosure via posts* as dependent variable. When operationalizing self-disclosure with the *horizontal width of self-disclosure via posts*, our hypothesis is not supported. Regarding the subsequent research question, we did not find interaction effects between the general decision-making tendency and a problematic social-networks-use in explaining self-disclosure via posts.

#### Results for Self-Disclosures via Profile Information as Dependent Variable

With respect to the general decision-making tendency, the *net score* had no significant influence on all dependent variables that were used to represent self-disclosures via profile information. This includes the *quantity of self-disclosure via profile* and the *horizontal width of self-disclosure via profile* (Table 5).

Similarly, the *s-IAT-SNS* sum score had no significant effect on any dependent variable representing self-disclosures via profile information (Table 5).

In the third step, the results overall revealed no considerable interaction effects. One significant but small interaction effect (*net score* × *s-IAT-SNS*: Δ*R*^2^ = 0.048, Δ*F* = 4.25, *p* = 0.042) was observed when taking the *quantity of self-disclosure via profile* as criterion (Model 1, Table 5). In this case, the overall model remained non-significant [*R*^2^ = 0.052, *F*(3,84) = 1.53, *p* = 0.213]. For all remaining interaction effects for self-disclosure via profile management we found: all Δ*R*^2^ ≤ 0.020, all Δ*F* ≤ 1.77, and all *p* ≥ 0.187.

In sum, our hypothesis is not supported when focusing on self-disclosure via profile information. With regard to the subsequent research question, the results revealed no considerable interaction effects between individual’s decision-making tendency and a problematic social-networks-use in explaining self-disclosure via profile information.

## Discussion

In the current study, we aimed at explaining privacy-related decisions on social media from a neurocognitive perspective by applying the theoretical lens of dual-process theories of decision making and by including a highly topical and relevant phenomenon, namely a problematic social-networks-use. By capturing participants’ actual self-disclosing behavior on the basis of their personal log-in on the most popular SNS (i.e., Facebook), we used a more objective measure to address self-disclosure on social media. Regarding our hypothesis and research question, the results revealed that posting a lot and disclosing much information within posts on Facebook is associated with the tendency to choose short-term (mainly rewarding) alternatives by neglecting long-term risks in situations that lack direct feedback about long-term outcomes. This can be interpreted in a manner that such a decision-making tendency (i.e., neglecting long-term risks in favor of immediate gratification, which indicates the tendency for impulsive processing) can lead to many disclosures, which in turn might increase the risk of negative long-term consequences. These results are in line with our assumption that a predominance of the impulsive system (which is responsible for short-sighted decisions) contributes to higher amounts of self-disclosures on social media as the accessibility of short- and long-term consequences differs here as well (Taddicken and Jers, 2011). Individuals might face an increased degree of uncertainty so that a strategic decision by weighing up risks and benefits as postulated in the *privacy calculus* (e.g., Dienlin and Metzger, 2016) is complicated and consequently, the impulsive system can predominantly lead the respective decision (see also Starcke and Brand, 2012). This might also explain why former studies noticed that users seem to be unhesitant when sharing large amounts of personal information online (Tufekci, 2008; Kokolakis, 2017). Moreover, other authors found that individuals in a highly emotional state often express their feelings or frustration and regret their posts afterward (Wang et al., 2013a), which indicates somehow impulsivity-driven and less deliberated posts. Although some users might also act primarily long-term oriented when disclosing personal information (e.g., to become more popular), these results strengthen the assumption that self-disclosure on social media can also be driven by short-term oriented decision making.

Besides the scarcity of feedback about possible long-term consequences, the specific environment that is provided by social media is likely to additionally support individuals to rely on automatic affect as it allows for the frequent experience of immediate rewards (Taddicken and Jers, 2011), which in turn can lead to the development of incentive salience (cf. Brand et al., 2019). This is also in line with our findings that a tendency toward a problematic social-networks-use led to an increased *quantity of self-disclosure via posts*, which (in case of this operationalization) supports our postulated hypothesis. When considering the I-PACE model by Brand et al. (2019), affective and cognitive responses to internal and external triggers (e.g., an increased attention toward an application’s pop-up message), can lead to the decision to open the application and post specific content, which then enables the experience of gratification (e.g., by getting immediate positive feedback via Likes). This can subsequently reinforce and increase reward expectancies. Consequently, being confronted with specific cues in future situations can enhance the anticipation of gratification, leading to further self-disclosures and gratification experiences which can end up in a problematic use of social media applications as the control over one’s behavior becomes challenging. Furthermore, when specific cues become more salient, individuals might react with an increased desire or even craving and might develop cue-related reductions in inhibitory control, leading to more habitual decisions to use those applications and to share personal information. Such a habituation might additionally be supported by a lack of feedback about possible long-term risks, which enhances the predominance of the impulsive system. Wang et al. (2013a, p. 1315) stated furthermore, that “when posting on Facebook becomes habitual, people rarely think about why they post things. […] Some users also did not think about the potential consequences of their postings.” So, in early stages of a problematic social-networks-use (Brand et al., 2019), learning processes (e.g., that creating posts can gratify one’s need for recognition) can foster impulsive processes and with increasing anticipation of gratification over time, more information is disclosed. Thus, individuals with an increased tendency toward a problematic social-networks-use are at risk of disclosing much personal information on social media, which is supported by the present results concerning self-disclosure via posts.

Referring back to our differentiation between posts and profile information, shortsighted decision-making tendencies and a problematic social-networks-use were found to be a predictor for self-disclosing behaviors in the course of posts but not regarding profile management. This might be traced back to the heterogeneity of posts and profile information: while posts enable users to frequently disclose and share personal content and thereby enhance the experience of feedback by others, profile information mainly encompasses rather stable aspects such as one’s birthday, current residence, sexual orientation or relationship status which are scarcely updated (cf. Strater and Lipford, 2008) and are thus less able to provide gratification. Thus, individuals might more likely be confronted with or more frequently experience short-term rewarding effects in the course of posts than in the course of profile management. Even if direct feedback about possible long-term consequences is lacking here as well, the probably less rewarding experiences together with the media informing that depositing sensitive information (e.g., one’s phone number or current residence) can be very dangerous and increases the probability of privacy intrusions might explain why the reflective system could be triggered here as well.

In contrast to the results regarding the *quantity of self-disclosure via posts* we found no significant effects of decision-making tendencies and problematic social-networks-use on the *horizontal width of self-disclosure*, neither when considering posts nor when focusing on profile information. This might imply that deciding who should be able to see the respective information is not necessarily impacted by impulsive decision-making tendencies. The possibility of negative long-term consequences associated with a specific audience could in some cases be equally salient compared to the expected rewards, thus reducing an imbalance between both systems (cf. Bechara, 2005; Starcke and Brand, 2012; Schiebener and Brand, 2015). For example, it could be that individuals have already experienced (albeit rather delayed) negative feedback by others for instance in the form of condescending comments regarding a post, leading to a more reflective audience selection in subsequently comparable situations. Thus, in this case, and with respect to the horizontal privacy, individuals might also act rationally, which is supported by the findings that user conceptualizations of privacy seem to be stronger related to horizontal privacy controls than to vertical (Quinn and Epstein, 2018). In contrast, feedback on a vertical level regarding negative long-term consequences (e.g., commercial exploitation) is hardly ever experienced, which can explain why individuals still disclose large amounts of personal information on social media and why we nevertheless observed the associations between the extent of self-disclosure and decision-making tendencies. Furthermore, that a problematic social-networks-use wasn’t a predictor in this context either might be because specific reward expectancies and anticipated gratification might be stronger associated with the content itself that is shared than with specific categories of audiences. However, if information was shared, the *quantity of self-disclosures via profile* was positively related to (a) the visibility “only for me,” (b) the visibility “for (certain) friends,” and (c) the visibility “for everybody (the public).” Further, the *quantity of self-disclosures via posts* was positively related to (a) “for (certain) friends” and (b) “for everybody (the public).” It follows that individuals in some cases restrict the accessibility of information on a horizontal level, but not in all cases. Further, restricting the access does not mean that they disclose less information, as the positive correlations show. That the contrary can be the case was also stated in other works (e.g., Stutzman et al., 2013).

Finally, decision-making tendencies and a problematic social-networks-use in interaction with each other did not explain further variance of individual’s self-disclosure as we did not find overall considerable interaction effects. Even if a preference for immediate rewards and more shortsighted behaviors was found in individuals with different IUD types (Schiebener and Brand, 2017), we did not find significant relations between the *net score* and the sum score of the *s-IAT-SNS* in this study. For self-disclosures on Facebook via posts it seems that (at least with regard to the current participants) general decision-making tendencies as well as an excessive social-networks-use can act as a predictor independently of the respective other. That means that on the one hand, individuals who tend to focus on short-term choices if immediate feedback about possible negative consequences is missing, are prone to disclose a lot of personal information on social media as they might not be able to compensate for the missing feedback. In this case, the general decision-making tendency can impact specific privacy-related behaviors on social media, even independently of an excessive usage. On the other hand, individuals who have developed a problematic use of social media applications have likely learned that using social media to disclose personal information can lead to immediate rewards (cf. Brand et al., 2019), resulting in increased expectancies and anticipation of gratification. In this case, the incentive salience of rewards might be a driving factor that leads to the disclosure of much information. In sum, the findings indicate that tendencies toward a problematic social-networks-use contribute to self-disclosing behaviors additionally to (and independently from) the general decision-making tendency to prefer immediate rewards by neglecting long-term risks. However, to further examine the relationship between both predictors in this context, research should focus more on context-specific decision-making tendencies. A problematic social-networks-use might be related to stimuli-specific decision-making deficits (cf. Brand et al., 2019) and not necessarily to decision-making deficits in general. By addressing social media related decision-making situations that lack feedback about long-term consequences, research could then further investigate possible interactions of both constructs in the context of online self-disclosures.

### Outlook and Implications

The results indicate that the general tendency to neglect long-term consequences in favor of immediate gratification contributes to the disclosure of much information within posts. This supports the assumption that not only reflective, but also impulsive processes play an important role in the context of online self-disclosures. Since direct feedback about possible long-term consequences regarding self-disclosures is (especially on a vertical level) missing and the experience of gratification and rewards is supported, rational decisions might be perturbed. Future studies need to focus more on self-disclosure in the course of posts as especially there may be an imbalance between the impulsive and reflective system. Moreover, posting content is a key function of many social media applications, especially of those increasingly used by younger individuals, such as Instagram, TikTok or Snapchat (Statista, 2019a). Here, personal content can extensively be used on both, a vertical (e.g., by the platform itself or third parties) and a horizontal level (by other users), while the effects on one’s privacy are mostly communicated in a rather hidden manner. Instead, such applications provide several features, such as predesigned filters or location information ready to be embedded, which enable quick, uncomplicated, and easy posts. This critical aspect is in line with former works stressing the impact of specific social media cues (e.g., Trepte, 2015) or website/application designs in general, as an “intentional use of certain design aspects in order to drive users into less deliberate, even misguided decisions” (Efroni et al., 2019, p. 356) can be observed in different online areas. Furthermore, as strong impulsions for a specific behavior can also result from internal stimuli (e.g., negative feelings, Fillmore, 2001; feelings of loneliness or boredom, Hofmann et al., 2009; Matook et al., 2015), situational and personal aspects should additionally be investigated in this context, similarly to further (possibly preventive) aspects such as privacy literacy including individual’s knowledge and skills (e.g., Bartsch and Dienlin, 2016; Büchi et al., 2017).

With regard to protective approaches, the relevance of feedback about potential long-term effects needs stronger consideration as providing such feedback can support individuals with impulsive tendencies to make more advantageous decisions (Müller et al., 2017). Accordingly, immediate feedback about possible long-term risks regarding currently disclosed personal information might support users in making more informed and conscious decisions, thus probably leading to less disclosures within their posts that involve the possibility of negative consequences. By using privacy nudges, Wang et al. (2013b) for example demonstrated that supporting information and feedback can encourage users to change privacy settings before they post something. The need for technical support is also posited by more recent works (Acquisti et al., 2017; Spottswood and Hancock, 2017; Aïmeur et al., 2019; Efroni et al., 2019), whereby research needs to find ways to support users without patronizing them and thus without creating reactance (Krämer and Schäwel, 2020). Additionally, research needs to critically investigate how privacy notices should be designed in order to be effective, for instance in terms of length and framing (e.g., Gluck et al., 2016), and how they could be made more adaptive to the user (e.g., Díaz Ferreyra et al., 2019).

Moreover, as individuals with a problematic social-networks-use seem to be prone to disclose much information within their posts, such emerging phenomena should be further targeted in research. Even though it has not yet been included in international classification systems, the inclusion of related phenomena such as gaming disorder and the reported prevalence rates for a problematic social-networks-use especially among young individuals and adolescents (e.g., European-wide prevalence rates up to 2.1%, Smahel et al., 2020; 2.6% in German adolescents, Wartberg et al., 2020) demonstrate the need for further research. This kind of overuse is by now almost a normal part of everyday life, more or less socially accepted, and legal (Turel et al., 2014). However, persons affected are likely to experience negative consequences in their everyday lives and are more likely to develop symptoms of depression (Keles et al., 2020). Further, based on this study’s results, tending to use social networks problematically could also result in privacy-related negative consequences due to increased self-disclosures. Since especially young individuals seem to be vulnerable to develop such problematic behaviors (Andreassen et al., 2016), the need for sufficient privacy protection mechanisms in order to prevent individuals from potentially risky self-disclosures on social media is again evident here. Further, improving specific competences (e.g., online self-regulative competences) appears to be pivotal to prevent problematic behaviors and should therefore be integrated in youth-tailored prevention/intervention programs (Ostendorf et al., 2020). The relevance of specific Internet-related skills for preventing risky and disadvantageous self-disclosures was also highlighted in other works (e.g., Büchi et al., 2017) and should therefore also be included in prevention/intervention programs. Vulnerable individuals should be supported in a way that they do not lose control over their social-networks-use and do not perceive these applications as only possibility to socially connect (e.g., via extensive self-disclosures), since this can end up in various negative consequences including privacy breaches (cf. Ostendorf et al., 2020).

Finally, some limitations have to be mentioned. This laboratory study used a convenience sample which is not representative for the entire population. Also, the measurement of self-disclosure is not entirely objective, since the classification of posts resulted from the participants’ own (subjective) evaluation/interpretation of their postings. To a certain extent, this might have been challenging for some participants and we therefore recommend to provide example posts in future studies. However, confronting participants with their own posts and provided profile information again when evaluating such aspects makes this approach still more objective and precise than common self-reports of behavior, which largely rely on participants’ recall and estimation. An aspect that was not specifically considered in this work is the specific content within posts and the sensitivity of the disclosed information. Including the quality of self-disclosure as a potential indicator of which disclosures might make individuals more vulnerable for privacy risks than others is important for further research. Future investigations should therefore try to include a vulnerability factor in the measure of self-disclosure, and also take into account both the vertical and horizontal dimension when defining the respective vulnerability level. Nevertheless, the results of this study contribute to the research field and thus appear worthwhile to be investigated further since there are first indications for underlying decision-making processes in the context of privacy-related decisions on social media. To even better understand these underlying processes, future studies should also put effort in developing a decision-making paradigm that is adapted for more context-specific, social media related decision situations. Additionally, recent research focuses on formal models of dual processes that allow for predictions of choice probability and response time (e.g., Loewenstein et al., 2015; Diederich and Zhao, 2019), which might also be applied to research on decision making in social media (and other contexts) in the future. Lastly, we focused on one specific social network (Facebook) to investigate privacy-related behaviors where participants created on average relatively few posts (about five posts in the last 3 months, even if some created up to 27). Given that other applications such as Instagram or TikTok live even more from the posts of their users than Facebook does, we assume that the effects will hold true for other platforms as well or might be even more pronounced. Nevertheless, further studies are needed to make informed statements on the transferability of the current findings to other social media applications.

### Conclusion

The current study showed that investigating privacy-related decisions on social media by applying dual-process theories of decision making helps to better understand seemingly paradoxical behaviors. The assumption of rationality as the basis of many theoretical approaches such as the privacy calculus or the Theory of Planned Behavior has to be critically questioned as users usually face a scarcity of feedback about possible long-term consequences and additionally can experience immediate gratification and benefits, which both can enhance impulsive decisions. The current findings indicate that individuals tending to prefer short-term gratifications by neglecting long-term risks are vulnerable to self-disclose a lot via posts on social media. Moreover, tending to use social media problematically can additionally increase individual’s self-disclosure via posts. Future studies need to follow up on these aspects and should develop protective mechanisms that enable users to make informed, conscious and more rational decisions on social media.

## Data Availability Statement

The datasets generated for this study are available on request to the corresponding author.

## Ethics Statement

The studies involving human participants were reviewed and approvedby the local Ethics Committee of the Department of Computer Scienceand Applied Cognitive Science at the Faculty of Engineering, University of Duisburg–Essen. Participants provided their written informed consent to participate in this study.

## Author Contributions

SO, SM, and MB contributed to the conceptualization, design, and methodology of the study. SO collected and analyzed the data, contributed to visualization, and wrote the first draft of the manuscript. SM contributed to visualization and validation, as well as reviewing/editing the manuscript. MB supervised the study and reviewed/edited the manuscript. All authors contributed to the manuscript and approved the submitted version.

## Conflict of Interest

The authors declare that the research was conducted in the absence of any commercial or financial relationships that could be construed as a potential conflict of interest.

## Abbreviations

CLT: Cards and Lottery Task
I-PACE model: Interaction of Person-Affect-Cognition-Execution model
IUD: Internet-use disorders
s-IAT: Short Internet Addiction Test
s-IAT-SNS: Short Internet Addiction Test modified for social networks
SNS: Social-networking sites.
